# SapC-DOPS nanovesicles: a novel targeted agent for the imaging and treatment of glioblastoma

**DOI:** 10.18632/oncoscience.122

**Published:** 2015-02-09

**Authors:** Víctor M. Blanco, Richard Curry, Xiaoyang Qi

**Affiliations:** ^1^ Division of Hematology and Oncology, Department of Internal Medicine, University of Cincinnati College of Medicine, Cincinnati, Ohio, USA; ^2^ Division of Human Genetics, Department of Pediatrics, Cincinnati Children's Hospital Medical Center, Cincinnati, Ohio, USA

**Keywords:** glioblastoma, phosphatidylserine, SapC-DOPS nanovesicles, tumor imaging, cancer therapy

## Abstract

Glioblastoma multiforme (GBM) is the most common malignant primary brain tumor. Classified by the World Health Organization (WHO) as grade IV astrocytoma, GBMs are extremely aggressive, almost always recur, and despite our best efforts, remain incurable.

This review describes the traditional treatment approaches that led to moderate successes in GBM patients, discusses standard imaging modalities, and presents data supporting the use of SapC-DOPS, a novel proteoliposomal formulation with tumoricidal activity, as a promising diagnostic imaging tool and an innovative anti-cancer agent against GBM.

## Strategies for newly diagnosed and recurrent glioblastoma treatment

It is estimated that more than 10,000 people in the United States will be diagnosed in the year 2015 with GBM, the most common malignant primary brain tumor [1]. Originally described in 1926 by Drs. Cushing and Bailey as “spongioblastoma multiforme” [2], pathological hallmarks defined at that time still apply today, and include increased cellularity, mitotic figures, neovascularization, and pseudopallisading necrosis. Although it appears heterogeneous under the microscope, these tumors were initially thought to represent a homogenous disease. Improved understanding of the underlying genetic and molecular profiling has dismissed this notion, supporting the belief that this is a very heterogeneous disease. For decades, the only proven beneficial adjuvant treatment following surgery was fractionated radiotherapy [3]. Numerous clinical trials combined chemotherapy and radiation, with disappointing results. By 2000, estimates were that chemotherapy added only a 6% absolute increase in 1-year survival for patients with newly diagnosed GBM [4]. Not until 2005 did the first phase III randomized controlled trial demonstrate statistically significant benefit from the addition of chemotherapy. Specifically, median overall survival increased from 12.1 months with radiation alone to 14.6 months with concurrent temozolomide and radiation [5]. These findings resulted in the FDA approval of temozolomide for newly diagnosed GBM, which remains the standard of care for patients today.

Options for treatment of recurrent disease traditionally have been limited. Repeat resection in select patients results in survival times ranging from 6 to 9 months [6]. Re-irradiation in the form of stereotactic radiosurgery yields results similar to surgery [7]. Additional chemotherapy has been equally as dismal. Platinum based chemotherapy, along with nitrosureas and other alkylating agents, result in a 25% progression-free survival at 6 months (PFS-6), with an overall survival (OS) of 6 to 9 months [8]. Moderate improvements in the care of GBM patients came in 2009 when the FDA approved bevacizumab, a monoclonal antibody that targets vascular endothelial growth factor (VEGF), as treatment for recurrent malignant gliomas, This approval was based on a number of phase II trials for recurrent GBM which demonstrated an improved overall response rate approaching 30-50% [9-12]. In addition to its potential survival benefits, bevacizumab's anti-edema properties offer an improvement in the quality of life for a number of patients. Bevacizumab monotherapy is the standard of care for recurrent GBM in the United States.

After bevacizumab's encouraging data for recurrent disease, other strategies were investigated, including two large phase III randomized controlled trials for newly diagnosed GBM. Unfortunately, overall survival time with bevacizumab was nearly identical when combined with standard concurrent chemoradiation or when used at recurrence [13, 14].

Tumor-treating fields (TTF), a type of low-intensity electromagnetic field therapy, have garnered significant interest in recent years. Although a randomized phase III trial showed no improvements in overall survival vs active chemotherapy, its lower toxicity and fewer adverse events prompted FDA approval of TTF for recurrent GBM in 2012 [15]. The concept of immunotherapy is blossoming throughout the field of oncology, including brain tumors. Dendritic cell vaccines for newly diagnosed GBM as well as vaccines targeting epidermal growth factor receptor (EGFRvIII) and heat-shock proteins in the newly diagnosed and recurrent settings are under investigation [16, 17]. A multitude of other approaches for treating GBM include but are not limited to oncolytic therapy, targeted therapies, pro-angiogenic pathway inhibition, and repeat radiation or chemotherapy in combination with bevacizumab [18].

## Imaging strategies

Limitations in neuroimaging present another challenge in the treatment of GBM. Quite often, it is difficult to distinguish between progression of tumor and treatment effect in the 3-6 months after completion of chemoradiation. Approximately 20-30% of patients will experience pseudoprogression in which magnetic resonance imaging (MRI) shows an increase in gadolinium enhancement and/or edema with no tumor growth [19-21]. Only with subsequent scans or surgery does it become evident whether the patient developed pseudoprogression or progressive tumor. MRI scans can continue to be confounding up to 12 months after chemoradiation. Specifically, 5-24% of patients with gliomas develop radiation necrosis, a severe inflammatory reaction to radiotherapy [19, 22, 23]. The difficulty in differentiating tumor from treatment effect has a significant impact on the patient's care.

Utilizing diffusion weighted imaging (DWI) and apparent diffusion coefficient (ADC) sequences on traditional MRI can improve the sensitivity and specificity to better differentiate pseudoprogression or radiation necrosis from true progression [24]. Accuracy can be further enhanced if MR-perfusion or spectroscopy based technology is employed [24, 25]. Positron emission tomography (PET) is an additional imaging tool in the armamentarium of treating neuro-oncologists, and yet like MR-based images, its sensitivity and specificity has limits [26-28].

In the following sections, the data presented will support the use of SapC-DOPS as both a unique diagnostic imaging tool and a treatment agent for GBM.

## SapC-DOPS targets phosphatidylserine, a pan-tumoral biomarker

Phosphatidylserine is an important membrane phospholipid that is synthesized by prokaryotic and eukaryotic cells, and it accounts for 3-10% of all cellular lipids [29]. In animal cells, a number of incompletely characterized lipid transporters (aminophospholipid translocases) work to maintain phosphatidylserine predominately located in the inner (cytosolic) leaflet of the plasma membrane. This asymmetry, which impacts diverse physiological processes [30], is lost in cells undergoing apoptosis (programmed cell death). In apoptotic cells, externalization of phosphatidylserine serves as an “eat me” signal that primes dying cells for macrophage engulfment [31].

Notably, many viable tumor and tumor-associated vascular cells present elevated levels of phosphatidylserine in their surface membranes [32-35]. Because it neither reflects apoptosis nor triggers phagocytosis by macrophages, the reason behind this phenomenon is uncertain [35]. Perhaps phosphatidylserine-exposing tumor cells are indeed primed for apoptosis but, because of their characteristic resistance, this process cannot be completed. However, recent evidence seems to indicate that enhanced phosphatidylserine exposure may confer adaptive advantages to cancer cells. It is known, for instance, that exposed phosphatidylserine plays a key role in prothrombin anchoring and activation of the coagulation cascade by platelets [36]; it is thus plausible that membrane surface phosphatidylserine (in conjunction with tissue factor) contributes to the pro-coagulant activity of some solid tumors, which favors their survival and dissemination [37, 38]. In addition, work in mice has suggested that melanoma metastasis is driven by phosphatidylserine-containing, tumor-derived microvesicles, which induce TGF-β expression and exert immunosuppression in macrophages [39]. Further evidence suggests that the latter may be an important aspect of tumor survival facilitated by the increased externalization of phosphatidylserine in tumor membranes. Moreover, many tumors express high levels of CD47, an anti-phagocytosis molecule that counteracts the pro-phagocytic signals transduced by phosphatidylserine binding [40].

**Figure 1:**
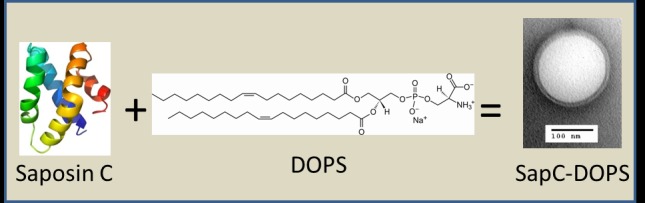
SapC-DOPS nanovesicles Saposin C (SapC) is an 80 amino acid, heat-stable, fusogenic protein that activates the lysosomal enzymes acid sphingomyelinase and acid beta-glucosidase, which catalyze the breakdown of sphingomyelin and glucosylceramide, respectively, into ceramide. At acidic pH, SapC binds to phosphatidylserine-enriched membranes (pKa ~5.3). When combined, SapC and dioleoylphosphatidylserine (DOPS) form stable, unilamellar proteoliposomes with anticancer activity. DOPS structure kindly supplied by Avanti Polar Lipids, Inc.

Work in our lab has led to the development of SapC-DOPS nanovesicles, a phosphatidylserine-targeting agent with tumoricidal activity (Fig. 1) [41]. Saposin C (SapC) is a natural protein found in lysosomes that binds membrane phosphatidylserine with high affinity and acts as a cofactor for the catalysis of glycosphingolipids [42]. Combining SapC and dioleoylphosphatidylserine (DOPS) leads to formation of stable proteoliposomes (~200 nm in diameter) that selectively bind and kill cancer cells *in vitro* and *in vivo* (Fig. 1). Studies in neuroblastoma and pancreatic cancer models showed that SapC-DOPS induces cancer cell apoptosis as a consequence of ceramide accumulation and caspase activation [43]. However, as discussed next, a different mechanism was found to mediate the killing of glioblastoma cells.

## Multimodal imaging of glioblastoma with SapC-DOPS

**Figure 2:**
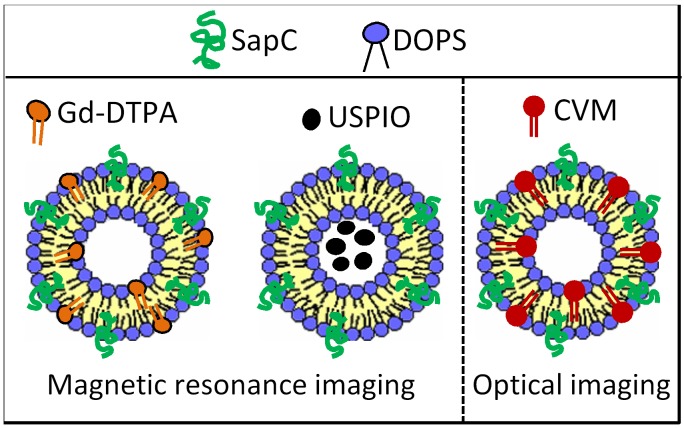
SapC-DOPS conjugates for GBM imaging MRI contrast agents (Gd-DTPA-BSA; USPIO) or lipophilic fluorescent probes, such as CellVue Maroon (CVM), can be embedded or encapsulated into SapC-DOPS for contrast-enhanced MRI or optical imaging.

**Figure 3:**
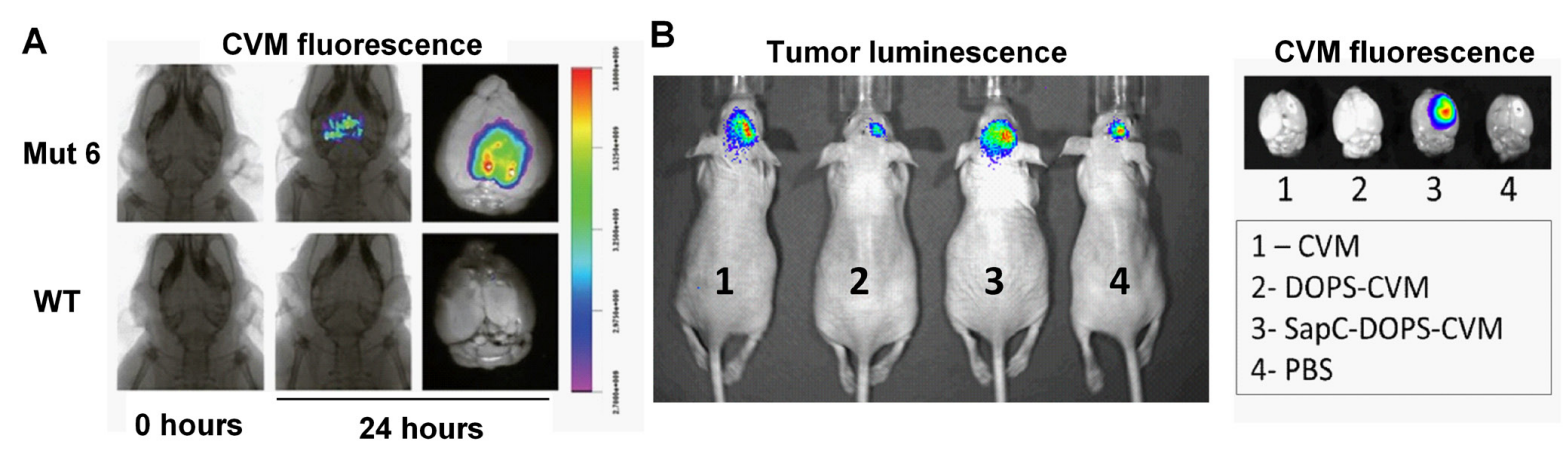
Optical imaging of GBM with SapC-DOPS-CVM A) Fluorescence imaging of a spontaneous GBM mouse model (Mut 6: GFAPcre; Nf1loxP/+; p53−/loxP; PtenloxP/+) and a wild-type mouse, 24 h after SapC-DOPS-CVM injection. B) *In vivo* tumor luminescence of orthotopic implants of human U87ßEGFR-Luc glioblastoma cells in athymic nude mice (left). Mice were injected i.v. with CVM, DOPS-CVM; SapC-DOPS-CVM or PBS and excised brains were imaged 24 h later (right).

**Figure 4:**
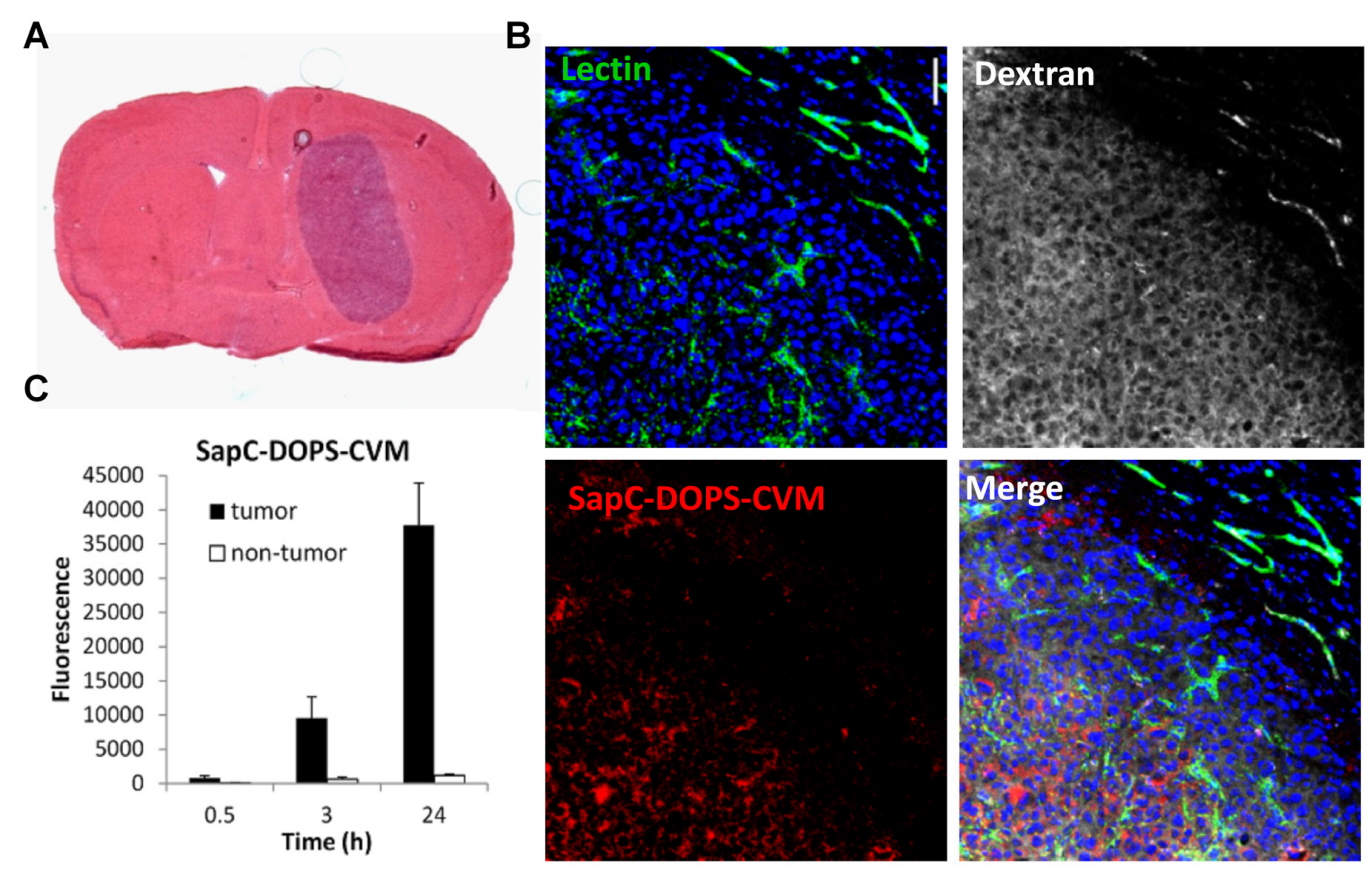
Intratumoral accumulation of SapC-DOPS-CVM A) Hematoxylin and eosin staining of a mouse brain section harboring a U87ßEGFR-Luc tumor. B) Confocal images of a GBM region and adjacent normal brain parenchyma shows specific intratumor accumulation of SapC-DOPS-CVM, 24 h after iv injection. Lectin-FITC and dextran-TRITC (MW 70 kDa) were injected before sacrifice to stain the vasculature and assess vascular permeability, respectively. C) Quantification of SapC-DOPS-CVM fluorescence from images like those shown in B.

By incorporating a lipophilic fluorescent dye (CellVue Maroon; CVM), or a paramagnetic gadolinium chelate (Gd-DTPA-BSA) into SapC-DOPS nanovesicles, we tested its tumor targeting capacity in preclinical models of GBM (Fig. 2). Using optical imaging, we showed that fluorescently labeled SapC-DOPS (SapC-DOPS-CVM) nanovesicles effectively targeted both spontaneous and xenografted (human) GBM in mice (Fig. 3). Histological analysis revealed that SapC-DOPS bound GBM vasculature, crossed the blood-brain barrier, and accumulated within tumors. In contrast, minimal signal was observed in the normal (non-tumoral) brain parenchyma (Fig. 4) [44, 45]. Importantly, since nanovesicles devoid of SapC (i.e., DOPS-CVM) do not effectively accumulate within GBM, the ability of SapC-DOPS to target GBM cells is not related to the increased permeability of tumor vessels (Fig. 4B) [45]. Instead, the selectivity towards tumor phosphatidylserine has been defined by showing that masking exposed phosphatidylserine in tumor cells either pre- or post-implantation greatly attenuates SapC-DOPS binding to GBM *in vivo* [44, 45].

These studies highlight the ability of SapC-DOPS to specifically target diverse GBMs in animal models and provide proof of principle for the use of fluorescently labeled SapC-DOPS in GBM imaging. Although translation to the clinical setting would require further advances in imaging technology, it may be a useful option in image-guided surgery for GBM resection.

**Figure 5:**
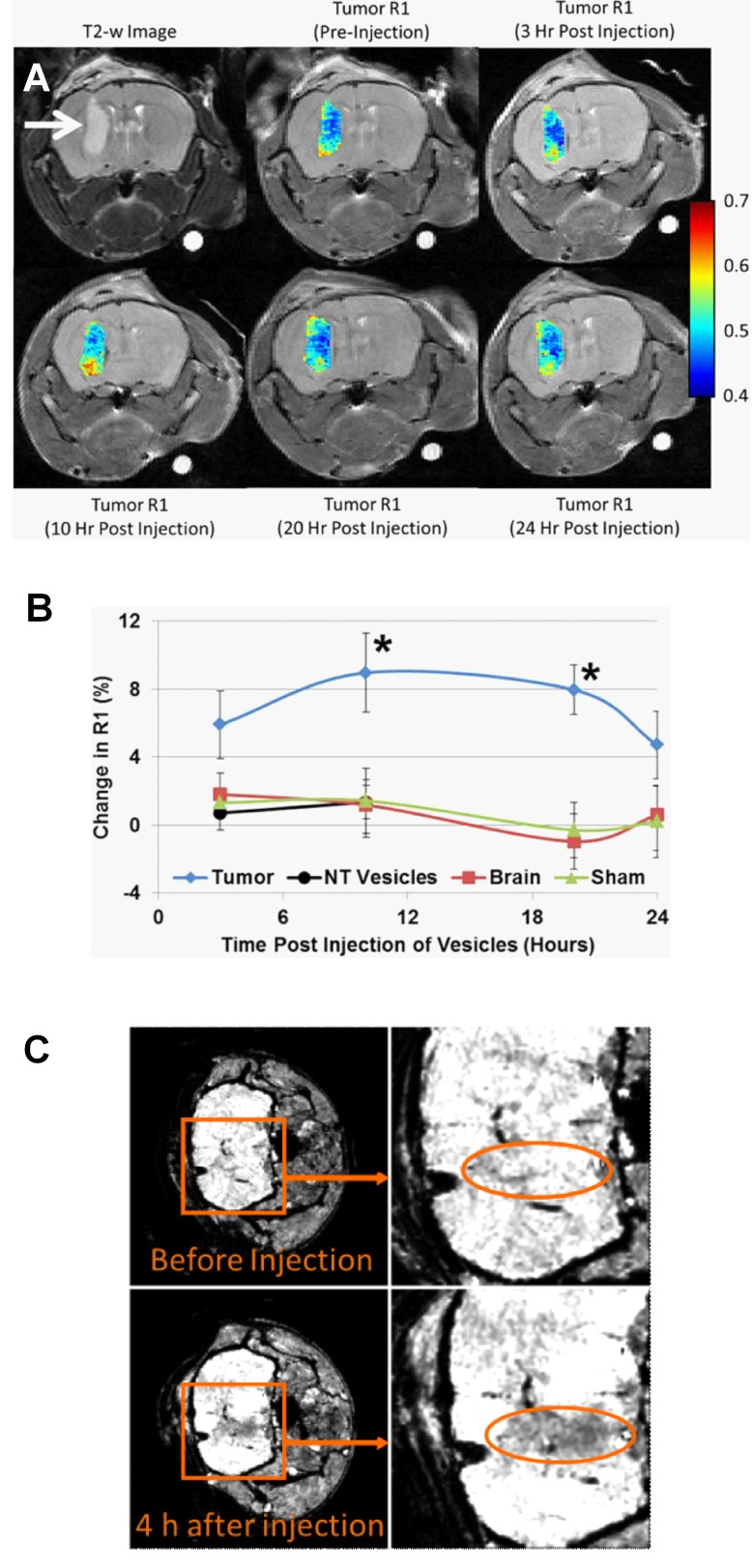
MRI of GBM with paramagnetic SapC-DOPS A) Changes in longitudinal relaxation rate (R1) in a mouse GBM after a single i.v. injection with Gd-DTPA-BSA/SapC-DOPS. A T2-weighted image (upper left) provides clear definition of the tumor (arrow). The color-coded R1 values show progressive increase with peak reached 10 h post injection. B) Percent change in R1 in the tumor, contralateral normal brain, and sham brain after injection of Gd-DTPA-BSA/SapC-DOPS nanovesicles, and R1 change in the tumor after injection of non-targeted (NT) vesicles (lacking SapC). At 10 and 20 h post-injection, the increase in R1 measured for Gd-DTPA-BSA/SapC-DOPS was significantly higher in the tumor (9.0% and 7.9%) compared with the normal brain, sham brain, or the tumor signal from non-targeted vesicles (<1.4%; *p<0.05). C) High-resolution MRI (7T) of a glioma in a nude mouse *in vivo* after a single i.v. injection with SapC-DOPS-USPIO. Negative contrast enhancement is observed in T2*-weighted images, 4 hours after i.v. injection of SapC-DOPS-USPIO (250 μl containing ∼22 μg iron).

Contrast-enhanced MRI with gadolinium (Gd^3+^), a technique widely used to evaluate brain lesions, reflects a non-specific increase in vascular permeability and is therefore restricted in its ability to provide guidance in the diagnosis and prognosis of gliomas [46]. Recently, we reported the use of paramagnetic SapC-DOPS nanovesicles as a targeted, T1-weighted contrast agent for MRI of GBM in the mouse brain (Fig. 2). Vesicles were formulated by addition of a lipophilic Gd^3+^ chelate, Gd^−^ DTPA-bis(stearylamide) (Gd-DTPA-BSA), and then tested in mice with orthotopic GBM tumors induced by injection of human U87ßEGFR-Luc cells [47]. In a previous study, we encapsulated ultra-small superparamagnetic iron oxide (USPIO; ferumoxtran-10) into SapC-DOPS for MRI of neuroblastoma [48]; pilot studies also showed the ability of this formulation for MRI of GBM in mice [49]. The results from these experiments are exemplified in Fig. 5. Our studies show that paramagnetic SapC-DOPS nanovesicles may be of greater value over conventional Gd^3^ probes as targeted contrast agents for GBM diagnosis and assessment in the clinical practice.

## Therapeutic actions of SapC-DOPS against GBM

**Figure 6:**
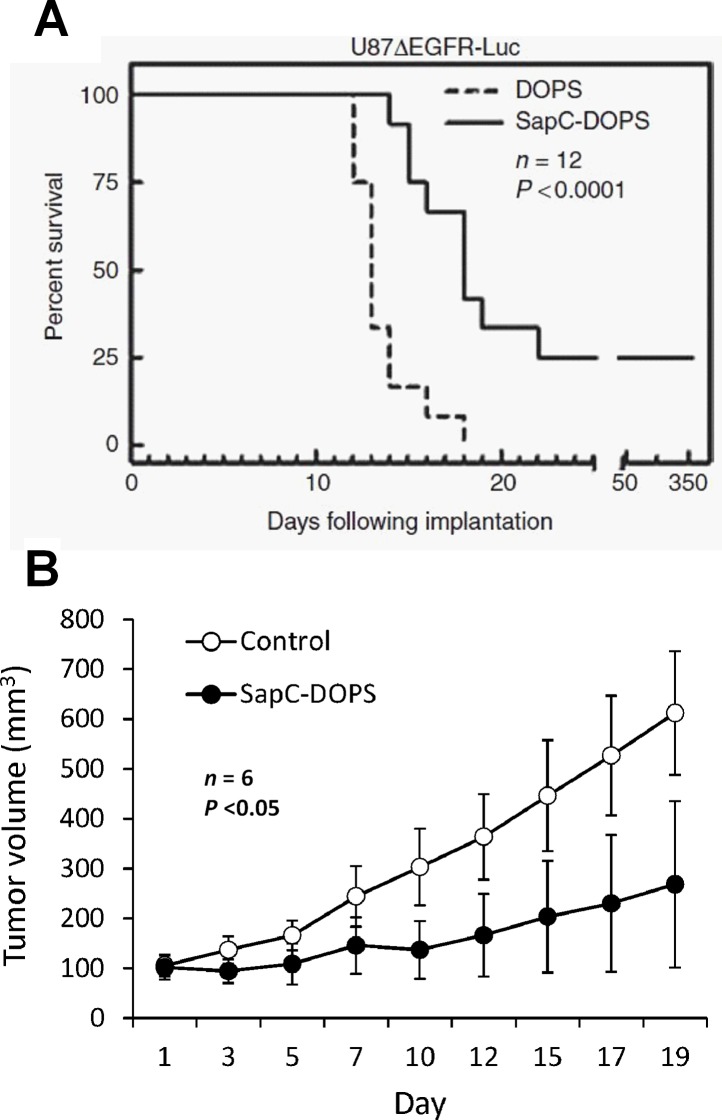
Therapeutic effects of SapC-DOPS on GBM mouse models A) Kaplan-Meier survival curve of mice bearing orthotopic human U87ßEGFR GBM. Mice were treated (i.v. tail injections) with SapC-DOPS or DOPS on the following days post-tumor implantation: 4–11, 13, 15, 17, 19, 22, 25, 28, and 31. B) Tumor volume measurements in mice bearing subcutaneous, human U87-MG GBM xenografts. Once tumors reached a mean volume of 100 mm3, mice received tail vein injections with a saline solution (Control) or SapC-DOPS daily for 7 days, and then every 2 days for 10 days.

Alterations in epidermal growth factor receptor (EGFR) signaling are very common in GBM and contribute importantly to its malignancy. We assessed the therapeutic efficacy of SapC-DOPS in preclinical models of GBM with or without EGFR alterations. As shown in Fig. 6, significant survival benefits were observed in mice bearing orthotopic GBMs carrying either the mutated, constitutively active EGFRvIII (human U87ßEGFR cells), or amplified EGFR (primary human X12 cells) [44]. In addition, a significant reduction in tumor growth was observed in xenografts of human U87-MG cells [45], which show low levels of wild-type EGFR [50]. These results suggest that the antitumor activity of SapC-DOPS is not related to the GBM's EGFR status. Interestingly, in a mouse model of GBM refractory to SapC-DOPS treatment, the combination of SapC-DOPS and temozolomide (the standard of care for GBM patients) had a strong synergistic effect that translated into a marked survival benefit compared with temozolomide alone [51]. We speculate that the induction of apoptosis by temozolomide increases tumor phosphatidylserine exposure, sensitizing GBM cells to the cytotoxic actions of SapC-DOPS.

The vascular endothelial growth factor (VEGF) inhibitor bevacizumab epitomizes the vast effort aimed at developing anti-angiogenic therapies for GBM. However, an unintended consequence of such therapies is tumor hypoxia, which promotes genetic instability and malignant progression. We recently reported that while SapC-DOPS had anti-angiogenic effects *in vivo*, the death of GBM cells *in vitro* was actually enhanced by hypoxia [44]. This phenomenon likely stems from the fact that hypoxia increases phosphatidylserine exposure in GBM cells, thus enhancing SapC-DOPS binding and toxicity. Taken together, these results suggest that SapC-DOPS, alone or in combination with apoptosis-inducing or anti-angiogenic drugs, may be a valuable therapeutic option for GBM patients.

## Mechanisms mediating SapC-DOPS-induced GBM cell death

Studies performed in a variety of cancer cells and animal tumor models indicated that SapC-DOPS anti-tumor effects are mediated by ceramide accumulation and caspase-dependent apoptosis [41, 43]. However, GBM cells are notoriously resistant to apoptosis. Studies in several GBM cell lines and primary GBM neurosphere cultures revealed that SapC-DOPS toxic effects were indeed unrelated to apoptosis, but instead involved lysosomal damage and necrotic cell death. Evidence included induction of lysosomal membrane permeability (LMP), demonstrated with lysosome-targeting fluorochromes, decreased glycosylation of the lysosome-associated membrane protein 1, reduced levels of mature cathepsin D, and increased levels of pro/preprocathepsin D [51]. GBM cell death was independent of p53, which was shown to confer resistance to radiation-induced apoptosis in U87 GBM cells by enhancing the degradation of ceramide [52]. The latter, in turn, has been shown to bind and activate cathepsin D [53]. GBM neurosphere viability could be significantly rescued by an acid ceramidase inhibitor, which prevented the hydrolysis of ceramide into sphingosine, a potent inducer of apoptotic and necrotic cell death through LMP [54]. This suggests that sphingosine is a key factor underlying SapC-DOPS-induced GBM cell death. Interestingly, SapC-DOPS treatment also induced significant autophagy signaling through the activation of c-Jun N-terminal kinases (JNK), although blockade of this phenomenon did not prevent cell death [51].

## CONCLUSIONS

The critical need for specificity and efficacy in cancer treatment is a consequence of both the inherent aggressiveness and recalcitrant nature of malignant tumors and the plethora of adverse side effects that result from off-target actions of current anticancer therapies. Identification of novel cancer biomarkers, namely molecules or genetic variations uniquely or predominately expressed by tumor cells, has not only predictive value but is the basis upon which new targeted therapies are continuously designed and eventually tested in clinical trials. With hundreds of clinical trials failing to provide significant breakthroughs and high rates of recurrence observed with current therapies, glioblastoma is paradigmatic of the challenge presented by highly malignant neoplasias [55]. SapC-DOPS nanovesicles target a ubiquitous tumor membrane biomarker, phosphatidylserine, and exert GBM cell death *in vitro* and *in vivo*. The vesicles selectively bind to tumor blood vessels and cross the compromised blood-brain barrier (i.e. blood-brain-tumor barrier), accumulating in GBMs of diverse origins and molecular characteristics. They present an excellent safety profile, with no adverse effects or organ toxicity being observed in studies conducted in mice [41]. Moreover, SapC-DOPS offers an optimal platform for further functionalization with imaging agents or anticancer drugs to improve its targeting and cytotoxic capabilities. Upcoming clinical trials conducted at the University of Cincinnati will evaluate the feasibility of using SapC-DOPS for GBM treatment.

## Abbreviations

SapC: Saposin C
DOPS: dioleylphosphatidylserine
GBM: glioblastoma multiforme
EGFR: epidermal growth factor receptor
VEGF: vascular endothelial growth factor (VEGF)
CVM: CellVue Maroon
LMP: lysosomal membrane permeability
JNK: c-Jun N-terminal kinases.
